# Toxic anterior segment syndrome following trabeculectomy with mitomycin C

**DOI:** 10.3205/oc000225

**Published:** 2023-09-29

**Authors:** Helen Ginger-Eke, Chimdia Ogbonnaya, Annamalai Odayappan, Jude Shiweobi

**Affiliations:** 1 Department of Ophthalmology, Ebonyi State University, Abakaliki, Nigeria; 2 Department of Ophthalmology, Alex Ekwueme Federal University Teaching Hospital, Abakaliki, Nigeria; 3 Department of Ophthalmology, Aravind Eye Hospital, Pondicherry, India

**Keywords:** TASS, trabeculectomy, complications, mitomycin C

## Abstract

**Objective:**

Toxic anterior segment (TASS) is a rare acute sterile anterior segment inflammation that typically develops within 12 to 24 hours after an anterior segment surgery. The purpose of this case report is to alert surgeons to the possibility of this complication following any anterior segment surgery, including trabeculectomy, and to highlight the possible etiologies and measures to prevent it.

**Patient and method:**

A 58-year-old male glaucoma patient was initially managed medically for primary open angle glaucoma with antiglaucoma medications. There was rapidly progressive glaucomatous optic nerve damage in his left eye within the following year, despite the use of antiglaucoma medications, hence the need for trabeculectomy.

**Result:**

The post-operative condition of the patient’s eye was stormy with diffuse limbus-to-limbus corneal edema and profound Descemet’s membrane folds, among other features of TASS, with associated deteriorating visual acuity.

**Conclusion:**

Although there is no documented report of TASS following trabeculectomy with mitomycin C, surgeons should be alerted to this possibility. Preventive measures include extreme care to avoid errors while preparing and administering diluted solutions, especially medications that are administered into the intracameral space.

## Introduction

Toxic anterior segment (TASS) is an acute sterile anterior segment inflammation that develops after anterior segment surgery, typically presenting within 12 to 24 hours [1].

Clinical manifestations of TASS vary widely. This is due to the anecdotal report of cases [2], [3], [4], [5], [6], [7]. Its hallmarks are minimal or no pain, pronounced cellular and fibrinous anterior chamber reaction, and diffuse limbus-to-limbus corneal edema secondary to damage from a toxic insult to the endothelial cell layer without posterior segment involvement [8].

Many substances have been reported to cause TASS, for example residues left behind by items used during cleaning and sterilization of surgical instruments, irrigating solutions with incorrect pH, osmolarity or ionic composition, preservatives, stabilizing agents, denatured ophthalmic viscosurgical devices, endotoxins, heavy metals, intraocular medications at toxic doses, and ointments [9], [10], [11], [12].

Apart from cataract surgery, TASS has been reported after anterior phakic intraocular lens (p IOL) implantation [13], penetrating keratoplasty [14], deep anterior lamellar keratoplasty [15], and triple Descemet’s stripping automated endothelial keratoplasty [16].

To the best of our knowledge, there is no report of TASS after trabeculectomy with mitomycin C in the literature. This report aims to heighten professional awareness of TASS regarding its possible occurrence in trabeculectomy surgery.

## Case description

A male patient, 58 years old with no systemic disease, presented to the glaucoma unit of Alex-Ekwueme Federal University Teaching Hospital, Abakaliki, with bilateral raised intraocular pressure about 3 years ago. He had no family history of glaucoma. On examination, his best corrected visual acuity was 6/5^–2^ on the right eye and 6/6^+1^ on the left. His intraocular pressure was 22 mmHg in the right eye and 34 mmHg on the left eye.

Slit-lamp examination on both eyes was within normal limits. The cornea was clear, and the peripheral anterior chamber depth was equal to the corneal thickness. Fundoscopy revealed a cup disc ratio of 0.2 in the right eye and 0.4 in the left eye. Pachymetry revealed a true intraocular pressure of 18 mmHg in the right eye and 29.7 mmHg in the left eye. Corneal thickness was 591 µm in the right eye and 612 µm on the left eye.

The patient was placed on latanoprost timolol combination eye drops once daily on the left eye. He also had oral acetazolamide tablets 250 mg every eight hours for ten days from his presentation onwards.

The follow-up of this patient a year later revealed a progression of optic nerve damage in the left eye with a cup disc ratio of 0.9 while the cup disc ratio in the right eye remained 0.2. However, intraocular pressure was 11 mmHg in the right eye and 18 mmHg in the left eye at the clinic visit. The patient was unable to do fundus photography. Central visual field analysis revealed a full field in the right eye and double arcuate defect. Therefore, he was planned for and underwent uneventful trabeculectomy surgery with mitomycin C in the left eye under local anesthesia. Postoperatively, the patient was placed on topical ciprofloxacin six-hourly and dexamethasone two-hourly for ten days, then gradually tapered off over three months, homatropine 2% eight-hourly and dexamethasone sodium ointment at night.

**Figure 1:**
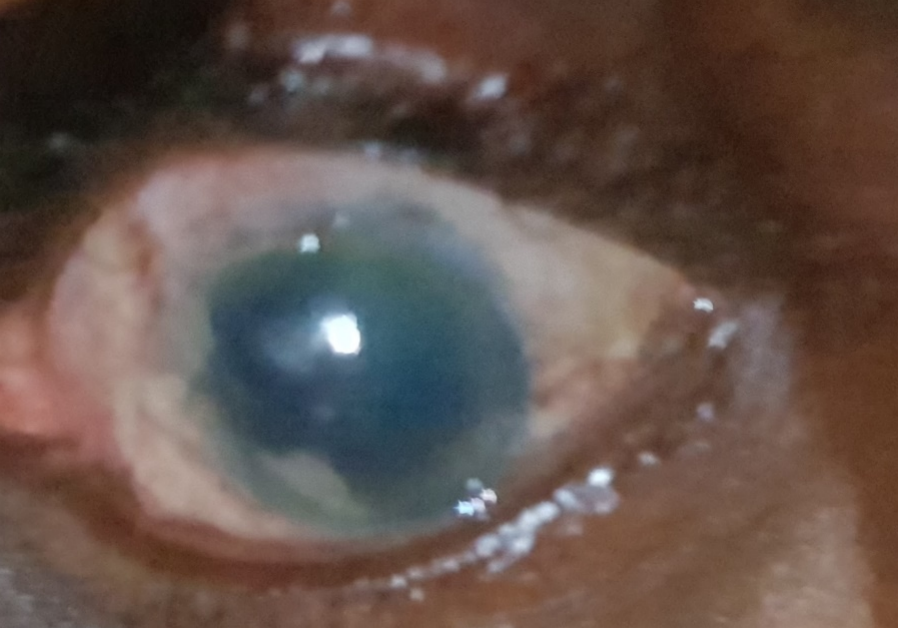
Left eye showing cornea edema

**Figure 2:**
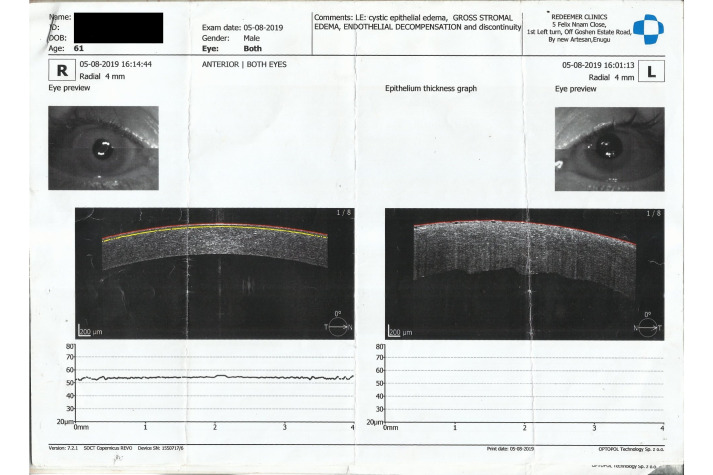
Anterior segment optical coherence tomography showing gross stromal edema

On the first post-operative day, the patient complained of reduced vision. The visual acuity was 6/36; there was a low diffuse bleb superiorly; the anterior chamber was formed but the patient had a generalized Descemet’s membrane folds with no pain; and the intraocular pressure was 15.6 mmHg. There were no cells, no flare, no fibrin, no hypopyon, the pupil was mid-dilated, the iris was normal, and no reaction in the vitreous was present. On the tenth day post-operatively, visual acuity reduced further to 1/60. There was an intense cornea edema (Figure 1 (Fig. 1)). He was treated with topical dexamethasone two-hourly, sodium chloride three-hourly, timolol twice daily, and a systemic oral acetazolamide 250 mg eight-hourly and prednisolone 20 mg twice daily for four days and tapered off over the next three days. Anterior segment OCT showed gross stromal edema (Figure 2 (Fig. 2)). During the follow-up visit in the fifth month, the patient had pain and subconjunctival hemorrhage which later resolved. However, cornea edema persisted, and bullous keratopathy developed, and the patient was referred to another center for penetrating keratoplasty, but he declined any other further intervention. Presently IOP is 8 mmHg and the patient is continued on topical hydroxypropyl methylcellulose eight-hourly.

## Discussion

Currently, there is no report of TASS after trabeculectomy in the literature. Our case report documents one of two patients operated on by the same surgeon on the same day. Four other eyes that had trabeculectomy on the same day did not develop TASS. In our analysis no probable cause was found. A likely factor could be inadvertent intracameral injection of medications used during surgery, such as gentamycin injection and mitomycin C injection. Koban et al. reported the first case of TASS following the inadvertent injection of a high dose of gentamicin into the anterior chamber [6]. One hour after surgery their patient had generalized Descemet’s membrane folds without pain. A similar finding was seen in our patient on the first post-operative day. Koban et al. also reported the presence of hyphema and iris hemorrhages. Our case had severe subconjunctival hemorrhage. This is a distinctive finding from previously reported cases.

## Conclusion and recommendations

There is no documented report of TASS following trabeculectomy with mitomycin C. Our case is a pointer that TASS could also occur after trabeculectomy surgery. Surgeons should be alerted to the danger of errors while preparing diluted solutions and as such should be careful while administering medications into the anterior chamber. Nurses in charge of surgical assistance should be properly educated on the topic. We suggest that syringes containing different medication for surgery should be properly labelled to avoid any mix-up while in the operating room.

## Notes

### Competing interests

The authors declare that they have no competing interests.
